# Geolocation with respect to personal privacy for the Allergy Diary app - a MASK study

**DOI:** 10.1186/s40413-018-0194-3

**Published:** 2018-07-16

**Authors:** D. Samreth, S. Arnavielhe, F. Ingenrieth, A. Bedbrook, G. L. Onorato, R. Murray, R. Almeida, M. A. Mizani, J. Fonseca, E. Costa, J. Malva, M. Morais-Almeida, A. M. Pereira, A. Todo-Bom, E. Menditto, C. Stellato, M. T. Ventura, D. Larenas-Linnemann, J-M. Fuentes-Pérez, Y. R. Huerta-Villalobos, A. A. Cruz, R. Stelmach, J. da Silva, R. Emuzyte, V. Kvedariene, A. Valiulis, I. Annesi-Maesano, I. Bosse, P. Demoly, P. Devillier, J. F. Fontaine, P. Kuna, B. Samolinski, L. Klimek, R. Mösges, O. Pfaar, S. Shamai, M. Bewick, D. Ryan, A. Sheikh, J. M. Anto, V. Cardona, J. Mullol, A. Valero, N. H. Chavannes, W. J. Fokkens, S. Reitsma, R. E. Roller-Wirnsberger, P. V. Tomazic, T. Haahtela, S. Toppila-Salmi, E. Valovirta, M. Makris, N. G. Papadopoulos, E. P. Prokopakis, F. Psarros, B. Gemicioğlu, A. Yorgancioglu, C. Bindslev-Jensen, E. Eller, I. Kull, M. Wickman, C. Bachert, P. W. Hellings, B. Pugin, S. Bosnic-Anticevich, R. E. O’Hehir, V. Kolek, M. Sova, K. Wehner, G. De Vries, M. van Eerd, D. Laune, J. Wittmann, J. Bousquet, P. Poncelet, the MASK study group, J. Bousquet, I. Agache, R. Almeida, R. Angles, I. Annesi-Maesano, J. M. Anto, S. Arnavielhe, E. Asayag, E. Bacci, C. Bachert, I. Baiardini, I. Baroni, B. A. Barreto, X. Basagana, A. Bedbrook, M. Bedolla-Barajas, K. C. Bergmann, L. Bertorello, M. Bewick, S. Bialek, T. Bieber, C. Bindslev-Jensen, L. Bjermer, A. Blua, M. Bochenska Marciniak, I. Bogus-Buczynska, S. Bosnic-Anticevich, I. Bosse, J. Bouchard, R. Bourret, V. Briedis, C. Bucca, R. Buonaiuto, M. T. Burguete Cabanas, D. Caiazza, D. Caillot, D. Caimmi, P. Camargos, G. Canfora, V. Cardona, A. M. Carriazo, C. Cartier, A. Carla Carvalho Coelho, G. Castellano, L. Cecchi, N. H. Chavannes, M. M. Ciaravolo, C. Cingi, A. Ciceran, L. Colas, E. Colgan, J. Coll, D. Conforti, J. Correia da Sousa, R. M. Cortés-Grimaldo, F. Corti, D. J. Costa, M. C. Costa Dominguez, A. L. Courbis, A. A. Cruz, A. Custovic, W. Czarlewski, C. Dario, J. da Silva, Y. Dauvilliers, G. De Carlo, F. De Blay, T. Dedeu, M. de Fátima Emerson, G. De Feo, M. H. Garcia Cruz, B. De Martino, P. Demoly, N. de Paula Motta Rubini, P. Devillier, G. De Vries, S. Di Capua Ercolano, N. Di Carluccio, G. Dray, R. Dubakiene, E. Eller, R. Emuzyte, J. M. Espinoza-Contreras, A. Estrada-Cardona, J. Farrell, A. Farsi, J. Ferreira de Mello, J. Ferrero, W. J. Fokkens, J. Fonseca, J. F. Fontaine, S. Forti, J. Garcia-Aymerich, J. L. Gálvez-Romero, C. I. García-Cobas, B. Gemicioğlu, R. Gerth van Wijk, M. Guidacci, J. Gómez-Vera, N. A. Guldemond, Z. Gutter, T. Haahtela, J. Hajjam, P. W. Hellings, L. Hernández, M. Illario, J. C. Ivancevich, E. Jares, G. Joos, J. Just, O. Kalayci, A. F. Kalyoncu, J. Karjalainen, T. Keil, N. Khaltaev, L. Klimek, M. L. Kowalski, I. Kull, P. Kuna, V. Kvedariene, V. Kolek, E. Krzych-Fałta, M. Kupczyk, P. Lacwik, D. Larenas-Linnemann, D. Laune, D. Lauri, J. Lavrut, M. A. Lessa, G. Levato, L. Lewis, I. Lieten, A. Lipiec, R. Louis, J. A. Luna-Pech, K. Maciej, A. Magnan, J. Malva, J. F. Maspero, E. Mathieu-Dupas, A. L. Matos, O. Mayora, M. A. Medina-Ávalos, E. Melen, E. Menditto, J. Millot-Keurinck, M. A. Mizani, G. Moda, M. Morais-Almeida, F. F. Morato-Castro, P. Moura Santo, R. Mösges, A. Mota-Pinto, J. Mullol, A. Murraro, R. Murray, M. Nalin, M. Noguès, E. Novellino, L. Napoli, H. Neffen, R. E. O’Hehir, G. L. Onorato, S. Palkonen, N. G. Papadopoulos, G. Passalacqua, J. L. Pépin, A. M. Pereira, M. Persico, O. Pfaar, R. Picard, P. Poncelet, F. Portejoie, A. C. Pozzi, D. Price, E. P. Prokopakis, R. Puy, B. Pugin, M. Przemecka-Green, F. Raciborski, R. Rajabian-Soderlund, S. Reitsma, I. Ribeirinho, J. Rimmer, J. A. Rizzo, M. C. Rizzo, C. Robalo-Cordeiro, X. Rodo, S. Rodrigues Valle, M. Rodríguez-González, G. Rolla, R. E. Roller-Wirnsberger, A. Romano, M. Romano, N. Rosario, D. Ryan, J. Salimäki, B. Samolinski, D. Samreth, S. Shamai, A. Sheikh, M. Sierra, F. E. R. Simons, D. Solé, M. Sorlini, O. Spranger, C. Stellato A, R. Stelmach, J. Strozek, R. Stukas, M. Sutherland, A. Szylling, J. N. Tebyriçá, M. Thibaudon, V. Tibaldi, A. Todo-Bom, S. Toppila-Salmi, P. Tomazic, U. Trama, M. Triggiani, M. Urrutia-Pereira, A. Valero, E. Valovirta, A. Valiulis, O. Vandenplas, M. van Eerd, T. Vasankari, A. Vatrella, M. T. Ventura, M. T. Verissimo, F. Viart, S. Williams, M. Wagenmann, M. Westman, M. Wickman, P. Wroczynski, A. Yorgancioglu, E. Zernotti, T. Zurbierber, C. Zubrinich, A. Zurkuhlen

**Affiliations:** 1 Kyomed, Montpellier, France; 2 Selbstregulierung Informationswirtschaft eV, Berlin, Germany; 3 MACVIA-France, Fondation partenariale FMC VIA-LR, Montpellier, France; 4 MedScript Ltd, Dundalk, Co Louth, Ireland; 5 Center for Health Technology and Services Research- CINTESIS, Faculdade de Medicina, Universidade do Porto; and Medina, Lda, Porto, Portugal; 6 Asthma UK Centre for Applied Research, Centre of Medical Informatics, Usher Institute of Population Health Sciences and Informatics, The University of Edinburgh, Edinburgh, UK; 7 UCIBIO, REQUINTE, Faculty of Pharmacy and Competence Center on Active and Healthy Ageing of University of Porto (Porto4Ageing), Porto, Portugal; 8 Institute of Biomedical Imaging and Life Sciences (IBILI), Faculty of Medicine, Ageing@Coimbra EIP-AHA Reference Site, University of Coimbra, Coimbra, Portugal; 9 Allergy Center, CUF Descobertas Hospital, Lisbon, Portugal; 10 Allergy Unit, CUF-Porto Hospital and Institute; Center for Research in Health Technologies and information systems CINTESIS, Universidade do Porto, Porto, Portugal; 11 Imunoalergologia Centro Hospitalar Universitário de Coimbra and Faculty of Medicine, University of Coimbra, Coimbra, Portugal; 12 CIRFF, Federico II University, Naples, Italy; 13 Department of Medicine, Surgery and Dentistry “Scuola Medica Salernitana”, University of Salerno, Salerno, Italy; 14 Unit of Geriatric Immunoallergology, University of Bari Medical School, Bari, Italy; 15 Center of Excellence in Asthma and Allergy, Hospital Médica Sur, México City, Mexico; 16 Mexico City, Mexico; 17 ProAR – Nucleo de Excelencia em Asma, Brasil and WHO GARD Planning Group, Federal University of Bahia, Salvador, Brazil; 18 Pulmonary Division, Heart Institute (InCor), Hospital da Clinicas da Faculdade de Medicina da Universidade de Sao Paulo, Sao Paulo, Brazil; 19 Department of Internal Medicine and Allergy Clinic of Professor Polydoro Ernani de São Thiago University Hospital, Federal University of Santa Catarina (UFSC), Florianopolis, SC Brazil; 20 Clinic of Children’s Diseases, Faculty of Medicine, Vilnius University, Vilnius, Lithuania; 21 Faculty of Medicine, Vilnius University, Vilnius, Lithuania; 22 Clinic of Children’s Diseases, and Institute of Health Sciences, Department of Public Health, Vilnius University Institute of Clinical Medicine, Vilnius, Lithuania; 23 European Academy of Paediatrics (EAP/UEMS-SP), Brussels, Belgium; 24 Epidemiology of Allergic and Respiratory Diseases, Department Institute Pierre Louis of Epidemiology and Public Health, Medical School Saint Antoine, INSERM and Sorbonne Université, Paris, France; 25 Allergist, La Rochelle, France; 26 Department of Respiratory Diseases, Montpellier University Hospital, Montpellier, France; 27 UPRES EA220, Pôle des Maladies des Voies Respiratoires, Hôpital Foch, Université Paris-Saclay, Suresnes, France; 28 Allergist, Reims, France; 29 Division of Internal Medicine, Asthma and Allergy, Barlicki University Hospital, Medical University of Lodz, Lodz, Poland; 30 Samolinski. Department of Prevention of Envinronmental Hazards and Allergology, Medical University of Warsaw, Warsaw, Poland; 31 Center for Rhinology and Allergology, Wiesbaden, Germany; 32 Institute of Medical Statistics, and Computational Biology, Medical Faculty, University of Cologne, Cologne, Germany; 33 CRI-Clinical Research International Ltd, Hamburg, Germany; 34 Department of Otorhinolaryngology, Head and Neck Surgery, Universitätsmedizin Mannheim, Medical Faculty Mannheim, Heidelberg University, Heidelberg, Germany; 35 iQ4U Consultants Ltd, London, UK; 36 Woodbrook Medical Centre, Loughborough, UK; 37 Honorary Clinical Research Fellow, Allergy and Respiratory Research Group, Usher Institute of Population Health Sciences and Informatics, University of Edinburgh, Medical School, Edinburgh, UK; 38 ISGlobAL, Centre for Research in Environmental Epidemiology (CREAL), Barcelona, Spain; 39 IMIM (Hospital del Mar Research Institute), Barcelona, Spain; 40 CIBER Epidemiología y Salud Pública (CIBERESP), Barcelona, Spain; 41 Universitat Pompeu Fabra (UPF), Barcelona, Spain; 42 S Allergologia, S Medicina Interna, Hospital Vall d’Hebron, Barcelona, Spain; 43 Rhinology Unit & Smell Clinic, ENT Department, Hospital Clínic; Clinical & Experimental Respiratory Immunoallergy, IDIBAPS, CIBERES, University of Barcelona, Barcelona, Spain; 44 Pneumology and Allergy Department CIBERES and Clinical & Experimental Respiratory Immunoallergy, IDIBAPS, University of Barcelona, Barcelona, Spain; 45 Department of Public Health and Primary Care, Leiden University Medical Center, Leiden, The Netherlands; 46 Department of Otorhinolaryngology, Academic Medical Centre, Amsterdam, the Netherlands; 47 Department of Internal Medicine, Medical University of Graz, Graz, Austria; 48 Department of ENT, Medical University of Graz, Graz, Austria; 49 Skin and Allergy Hospital, Helsinki University Hospital, Helsinki, Finland; 50 Department of Lung Diseases and Clinical Immunology, University of Turku and Terveystalo allergy clinic, Turku, Finland; 51 Division of Infection, Immunity 1 Respiratory Medicine, University of Manchester, Manchester, UK; 52 Allergy Department, 2nd Pediatric Clinic, University of Athens, Athens, Greece; 53 Department of Otorhinolaryngology University of Crete School of Medicine, Heraklion, Greece; 54 Allergy Department Athens Naval Hospital, Athens, Greece; 55 Department of Pulmonary Diseases, Istanbul University, Cerrahpasa Faculty of Medicine, Istanbul, Turkey; 56 Department of Pulmonary Diseases, Faculty of Medicine, Turkey and GARD Executive Committee, Celal Bayar University, Manisa, Turkey; 57 Department of Dermatology and Allergy Centre, Odense Research Center for Anaphylaxis (ORCA), Odense University Hospital, Odense, Denmark; 58 Department of Clinical Science and Education, Södersjukhuset, Karolinska Institutet, Stockholm, Sweden; 59 Centre for Clinical Research Sörmland, Uppsala University, Eskilstuna, Sweden; 60 Upper Airways Research Laboratory, ENT Department, Ghent University Hospital, Ghent, Belgium; 61 Department of Otorhinolaryngology, Univ Hospitals Leuven, Leuven, Belgium; 62 Academic Medical Center, Univ of Amsterdam, Amsterdam, The Netherlands; 63 Euforea, Brussels, Belgium; 64 Woolcock Institute of Medical Research, University of Sydney and Sydney Local Health District, Glebe, NSW Australia; 65 Department of Allergy, Immunology and Respiratory Medicine, Alfred Hospital and Central Clinical School, Monash University, Melbourne, Victoria Australia; 66 Department of Immunology, Monash University, Melbourne, Victoria Australia; 67 Department of Respiratory Medicine, Faculty of Medicine and Dentistry, Palacky University Olomouc and University Hospital Olomouc, Olomouc, Czech Republic; 68 Fachbereich Biologie, Technische Universität, Darmstadt, Germany; 69 Peercode BV, Geldermalsen, The Netherlands; 70 INSERM U 1168, VIMA: Ageing and chronic diseases Epidemiological and public health approaches, Villejuif, France; 71 Université Versailles St-Quentin-en-Yvelines, UMR-S 1168, Montigny le Bretonneux, France; 72 LIRMM, Montpellier, France

**Keywords:** Anonymization, App, MASK, Rhinitis, Asthma

## Abstract

**Background:**

Collecting data on the localization of users is a key issue for the MASK (Mobile Airways Sentinel networK: the Allergy Diary) App. Data anonymization is a method of sanitization for privacy. The European Commission’s Article 29 Working Party stated that geolocation information is personal data.

To assess geolocation using the MASK method and to compare two anonymization methods in the MASK database to find an optimal privacy method.

**Methods:**

Geolocation was studied for all people who used the Allergy Diary App from December 2015 to November 2017 and who reported medical outcomes. Two different anonymization methods have been evaluated: Noise addition (randomization) and k-anonymity (generalization).

**Results:**

Ninety-three thousand one hundred and sixteen days of VAS were collected from 8535 users and 54,500 (58.5%) were geolocalized, corresponding to 5428 users. Noise addition was found to be less accurate than k-anonymity using MASK data to protect the users’ life privacy.

**Discussion:**

k-anonymity is an acceptable method for the anonymization of MASK data and results can be used for other databases.

## Background

MASK-rhinitis (Mobile Airways Sentinel networK for allergic rhinitis) is a patient- centered ICT (Information and Communication Technology) system [1]. A mobile phone app (the *Allergy Diary App)*, central to MASK, is available in 22 countries. It has been validated [2] and found to be an easy and effective method of assessing the symptoms of allergic rhinitis (AR) and work productivity [2–5]. MASK follows the checklist for the evaluation of Good Practices developed by the European Union Joint Action JA-CHRODIS (Joint Action on Chronic Diseases and Promoting Healthy Ageing across the Life Cycle) [6]. The major aims of MASK are to provide care pathways [7] in rhinitis and asthma multimorbidity [8] including a sentinel network using the geolocation of users [9] and to inform the App user of the pollen and/or pollution risk level in their area, by means of geolocation. Both of these functionalities are being developed.

### European data protection law

The European data protection law only applies to personal data, i.e. “any information relating to an identified or identifiable natural person; an identifiable natural person is one who can be identified, directly or indirectly, in particular by reference to an identifier such as a name, an identification number, location data, an online identifier or to one or more factors specific to the physical, physiological, genetic, mental, economic, cultural or social identity of that natural person” (Art. 4 para. 1 no. 1 GDPR) [10]. Data anonymization is a method of sanitization for privacy. Anonymization renders personal data “in such a manner that the data subject is not or no longer identifiable.” (Recital 26 GDPR) [11]. As anonymous or anonymized data lack identifiability, anonymization principally enables the sharing of data in a way that preserves privacy with minimal data loss.

In 2014, lacking a clear statement within the law, the European Commission’s Article 29 Working Party (WP29) stated, with regards to the Directive 95/46/EC [12], that geolocation information is not only personal data but also to be considered as an identifier itself [13, 14]. This WP29 finding has become indisputable as the General Data Protection Regulation now clearly states within its definition of personal data (Art. 4 para. 1 no. 1 GDPR) that location data serves as an identifier.

Processing personal data legally under the European Data Protection Law first requires an assessment of the applicable law. Under the framework of Directive 95/46/EC [12], the situation was complex as the Directive may be implemented differently by the Member States of the European Union. Depending on the context of processing, compliance with additional legislation may be required.

Processing personal data by means of an app, such as the Allergy Diary App, is under the Directive 95/46/EC [12] and Directive 2002/58/EC [15] as amended by the Directive 2009/136/EC [16].

Since May 2018, the situation has become more stringent as the General European Data Protection Regulation now applies and all general national provisions on processing personal data are being overruled by European Law. Directive 2002/58/EC [15], as amended by Directive 2009/136/EC [17], is currently being revised and will also be replaced by a Regulation.

Processing personal data lawfully therefore requires (Art. 4 GDPR) either the data subject’s consent or any other legal ground being applied. Principally, such processing is unequivocally necessary for the performance of the service or contract concerned. For electronic communication services, such as apps, Directive 2002/58/EC [15] as amended by Directive 2009/136/EC [16] provides additional requirements.

Data on a subject’s smart device may only be accessed further to consent, (Article 5 para. 3 Directive 2002/58/EC [15] as amended by Directive 2009/136/EC) [16]. Such consent for technical access has to be distinguished from the potential legal ground on processing personal data [18]. Given the high sensitivity of location data, as highlighted by the clarification in Article 4 GDPR and multiple Opinions of the WP20 such as 00461/13/EN WP202 and 0829/14/EN WP216, apps should only technically access and process location data after explicit consent. The processing of personal data under data protection law may however find its legal ground in Article 6 para. 1 lit. b or lit. f GDPR and therefore does not require individual data subject’s consent in all circumstances.

Publishing and sharing location data may however require the data subject’s consent. As consent creates additional burdens, the anonymization of such data seems an appropriate option in providing a service like the Allergy Diary App. Anonymization techniques are not all considered with the same level of confidence [13, 14]. The data of the *Allergy Diary* App are fully anonymous except for the data related to geolocation. The two main data anonymization processes, with differing strengths and weaknesses, are randomization and generalization [19, 20]. The randomization approach includes noise addition [21] and differential confidentiality [22]. k-anonymization [23–26] and its derivative processes (l-diversity [27] and t-closeness [28]) are the most widely accepted generalization approaches and are acceptable by WP 29.

## Methods

### Aim and design

In order to assess whether the anonymized geolocation level of the user of the MASK *Allergy Diary* is sufficient for the analyses planned, a study was set up including all people who had used the App from December 1st  2015 to November 30th  2017. Noise addition and k-anonymization were evaluated.

### Setting

The study included users from 22 countries who registered with the *Allergy Diary* App -available in 16 languages- through App stores. Geolocated data were retrieved from the users' smartphone and collected in every country where the App was available. This data retrieval was technically independent of the App.

### Participants

All consecutive users who registered with the *Allergy Diary* were included if they agreed to be geolocated. There were no exclusion criteria. Some of the users were clinic patients who had been asked by their physician to use the App. However, due to the anonymization of data, no specific information could be gathered, as previously described in detail [3, 4]. With their consent, five users (3 from Kyomed and 2 from Peercode) were considered as “testers” for the algorithm sensitivity analysis.

### Ethics

The Allergy Diary is CE1 registered [3, 4]. No ethical committee approval was needed for this study.

*Allergy Diary* App users agreed to be geolocated in the “terms of use” and “privacy policy” of the *Allergy Diary* App. Geolocation was optional, the user could allow it or not on his/her mobile phone and it could remove it at any time. Moreover, geolocation was not used in the data mining process and the phone IP was not recorded. Finally, the App functionalities were the same whether the user was geolocated or not.

### Outcomes reported in the allergy diary

Users assess their daily symptom control via the touchscreen functionality on their smart phone. They were invited to click on four consecutive visual analogue scales (VAS) (global evaluation, nasal, ocular, asthma) [3, 4].

### Geolocation of days reporting VAS

**Fig. 1:**
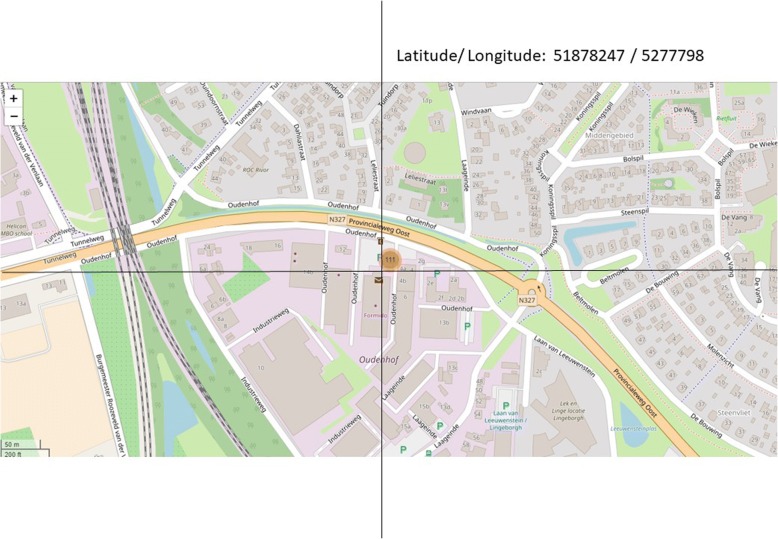
Geolocation using the ISO/TC211 standards (example: Peercode tester position)

ISO/TC 211 standards are currently being used to determine precise position and location by means of coordinates or geographic identifiers. The geolocation information appears as a set of two numbers corresponding to latitude and longitude (Fig. 1).

### Data analysis

We initially mapped the data in order to validate the fact that the geolocation data of the App users is an identifying process (https://folium.readthedocs.io/en/latest/). Experiments have been conducted by using the Folium Python Library and Leaflet maps (http://leafletjs.com/). Folium builds on the data wrangling strengths of the Python ecosystem and on the mapping strengths of the Leaflet. Folium visualizes data on an interactive Leaflet map. It enables the binding of data to a map for choropleth visualizations and Vincent/Vega visualizations as markers on the map. Clustering image pixels is an important image segmentation technique. We used the algorithm of Hou et al. [29] who combined DSets (dominant sets) and DBSCAN (Density-Based Spatial Clustering of Applications with Noise) to generate the clusters of arbitrary shapes without any parameter input.

We then assessed geolocation methods. A first experiment was to apply a random anonymization technique to the data set. We used noise addition by replacing the last two digits of the geolocation data by a zero value, which corresponds to blur geolocation data in a 10 by 10 km area [14].

A second set of experiments used a k-anonymization [26] method. k-anonymity allows the tolerable disclosure risk to be selected at the outset. For k-anonymity, the risk of identity disclosure is upper-bounded by 1/k. ε-Differential privacy can ensure a very low identity and disclosure (especially for small ε), but at the expense of an important utility loss. However, k-anonymity does not protect against attribute disclosure in general (e.g. if the values of a confidential attribute are very similar in a group of k records sharing quasi-identifier values). A common method for complying to the k-anonymity criterion is to generalize values in the quasi-identifiers by reducing their precision [30]. A release of data has the k-anonymity property if the information for each person contained in the release cannot be distinguished from at least k-1 individuals whose information also appears in the release. In our context, k stands for the minimal distinct days of symptoms. Obviously, the number of users must be greater than one, failing which it is still possible to identify this person. After a quantitative exploratory research, we gathered users at least by 2 and data at least by 5, which is a method accepted by the EU directive [8, 31].

For k-anonymity, we tested several values of *ε* on our data set. We tested data aggregation to get 5 minimum points from at least 2 users in a circle of 1 km of radius *(ε =* 1), 2.5 km (*ε =* 2.5 km), and 5 km (*ε =* 5 km). The haversine formula was used for the calculation of distances [32] as it determines the great-circle distance between two points on a sphere, given their longitude and latitude. This is the method recommended for calculating short distances by NASA’s Jet Propulsion Laboratory (https://www.jpl.nasa.gov).

Random anonymization techniques and k-anonymity were tested first of all on the five “testers” (with their consent) who used the App for over 200 days. The two techniques were then tested for confirmation on 518 users who declared more than 30 days of symptoms. The users declaring 7 to 15 days of VAS were given special focus, as they represent the targeted App users. Seven to 15 days of VAS allowed a sufficient number of events and appeared to be clinically relevant as most AR patients suffer from 7 to 15 days during the pollen season [33]. We did not study periods of between 15 and 29 days since the analyses of the database showed that there was a low number of users in this category (< 15%) and that the data were very heterogeneous (unpublished data). Finally, the two methods were tested on the users having declared only one day of VAS.

## Results

### Participants

From December 1st 2015 to November 30th 2017, 93,116 days of VAS were collected from 8535 users. 5428 (60.1%) users in 22 countries were geolocated, corresponding to 54,500 (58.5%) days (Tables 1 and 2). There was no major difference in the users’ geolocation rates between countries.

**Table 1:** World-wide repartition of geolocated days and users

Country	Nb of geolocalized data	Nb of geolocalized users
AT	1323	200
AU	354	45
BE	398	63
BR	2553	489
CA	66	11
CH	661	238
CZ	101	5
DE	4416	309
DK	485	54
ES	4043	283
FI	1305	167
FR	2206	316
GB	3168	278
GR	1583	89
IT	8500	706
LT	4073	211
MX	9707	496
NL	1304	218
PL	2300	300
PT	4819	810
SE	639	62
TR	496	78
Total	54,500	5428

**Table 2:** Online: repartition of VAS geolocated days and users included in the evaluation

VAS days of reporting	1	2–6	7–15	16–30	> 30
Number of users	2273	2311	234	92	518

### Geolocation of users

**Fig. 2:**
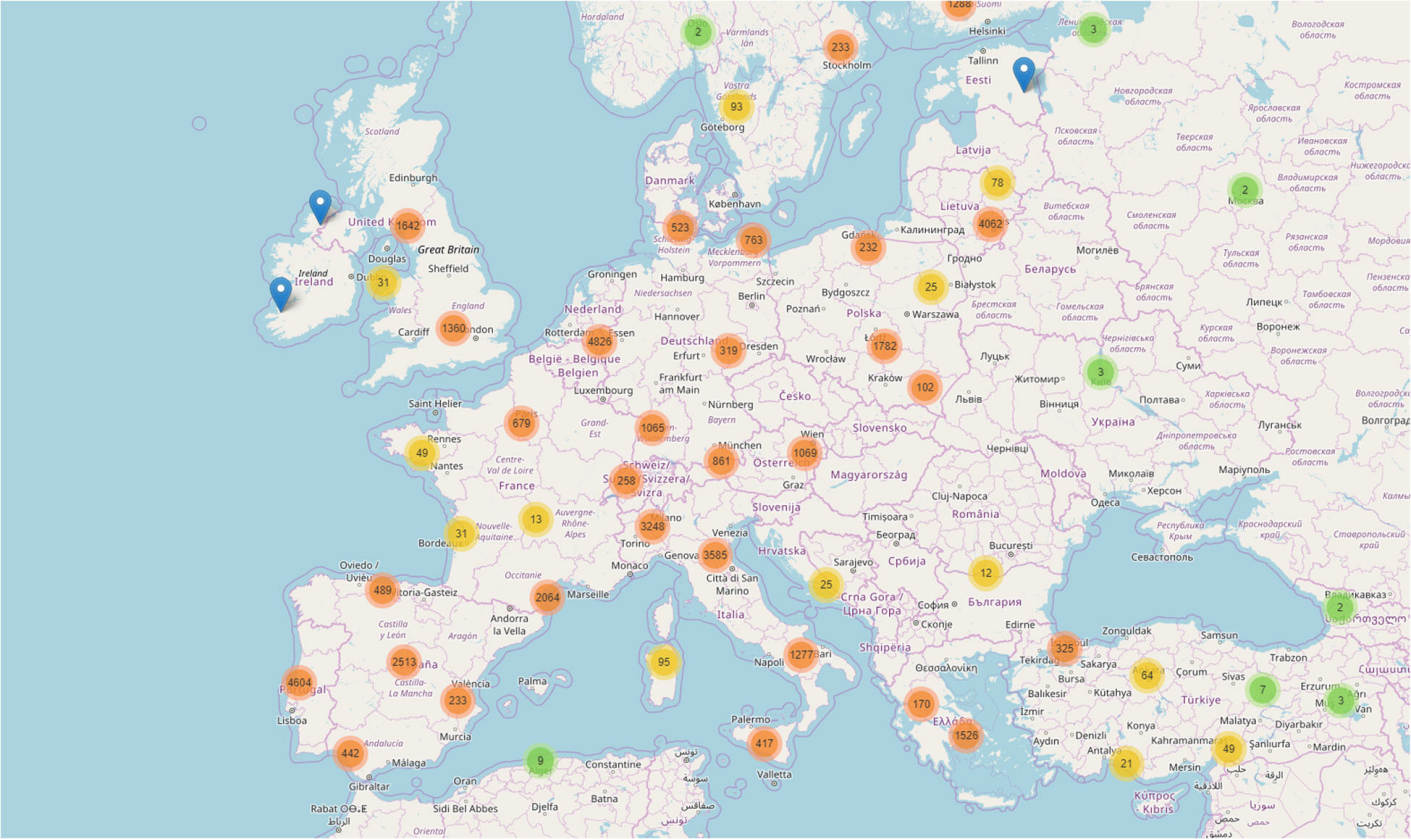
Geolocation of VAS days collected in Europe

**Fig. 3:**
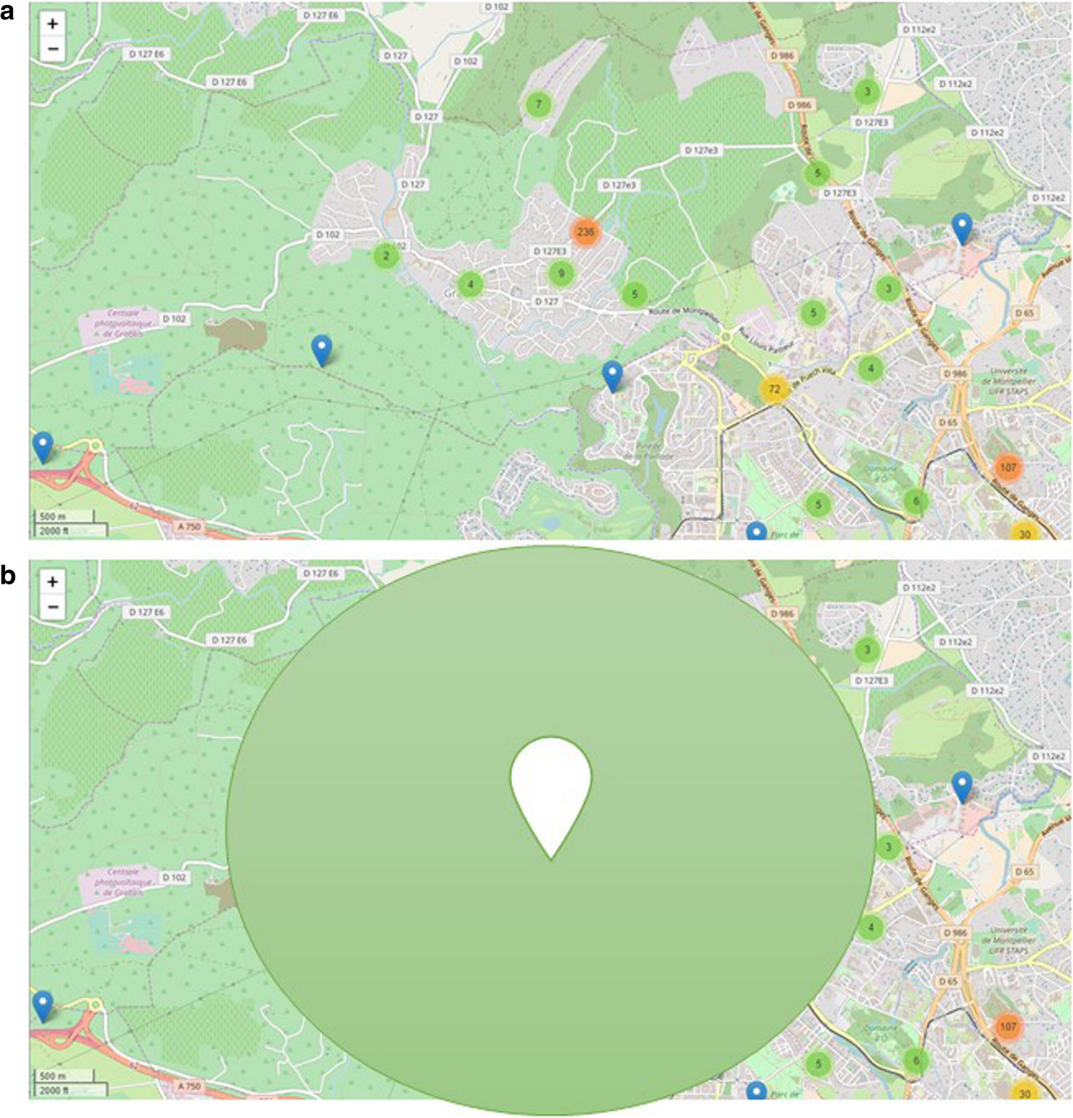
**a** VAS data of a single “tester”. **b** Geolocation data treatment with random method blurs, here only one user’s data

The geolocation of VAS days collected in Europe is reported in Fig. 2. The plot refers to days of symptoms. The color code is linked to the number of days reported. When zooming, we can associate days of symptoms to specific users (as described in Fig. 3), confirming that geolocation is an identifying process which is usable worldwide.

### Random anonymity

By including a zero value for the last two digits of the localization data, we could blur the location zone. When distinct users were close to each other (as in an urban zone), this process enabled the merging of different VAS data (of distinct users) in a single location zone. But in areas where only one user was using the App, miles away from the other closest user, the reported VAS data was linked only to this individual user at that location (Fig. 3). In these circumstances, the random method by noise addition did not enable the dissociation of the VAS data days from their owner. Figure 3a and b show data collected on one of the five “testers”.

Figure 3b shows the data collected from one of the five “testers” when the random method by noise addition has been considered. It shows that it does not enable the dissociation of  the VAS data days from their owner. Even if it is not possible to determine the precise location of the user, it is possible to infer his/her main location. The point is now located at the barycentre of all the previous locations. This method was tested on the three data subsets that were analyzed. We observed that 70% of the users declared symptoms within a circle of 1 to 9 km. This method is therefore not a de-identification method in our data set.

### Generalization approach using k-anonymity

The k-anonymity algorithm was tested on users according to the number of VAS they reported (Table 2).

**Fig. 4:**
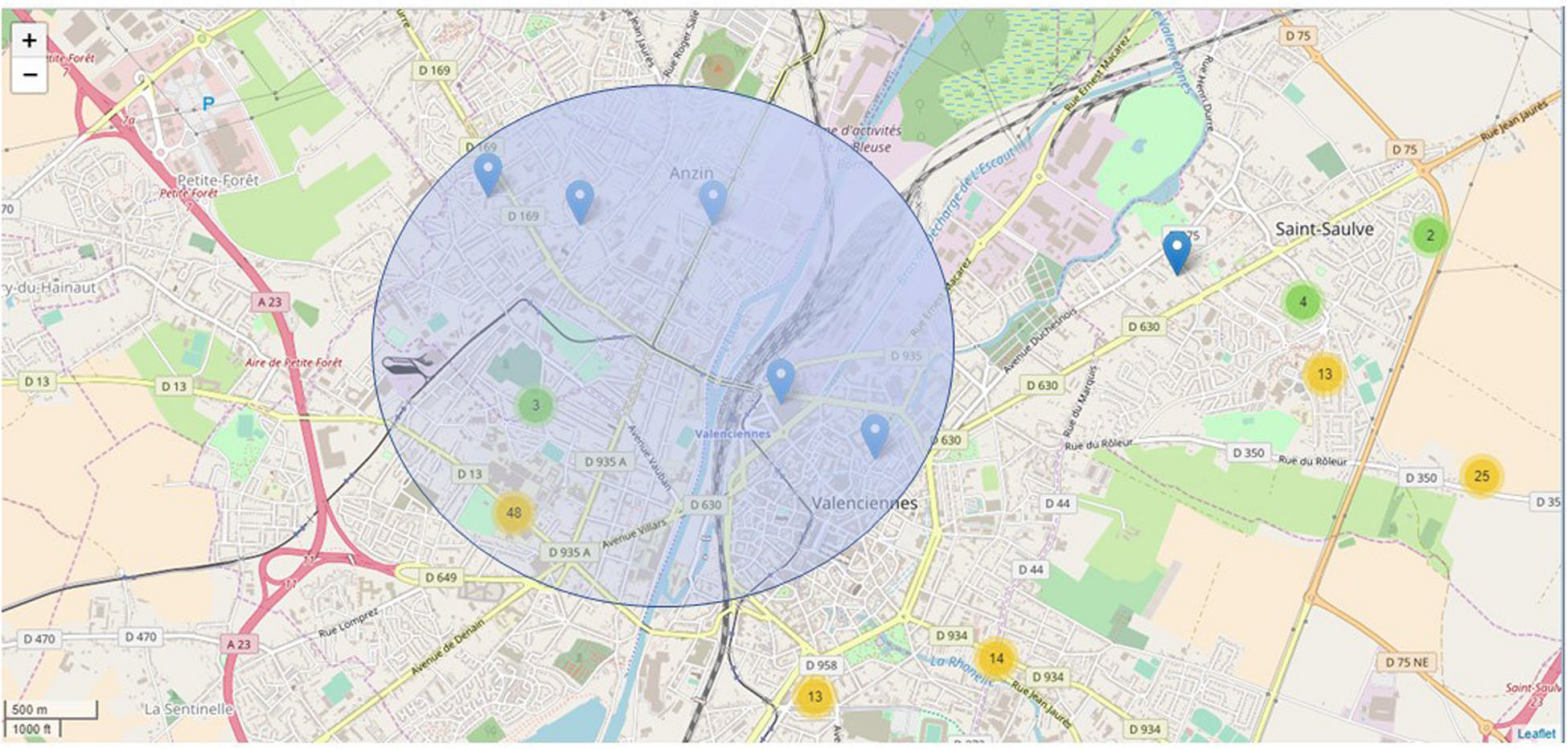
k-anonymity applied to users in Valenciennes (France)

The k-anonymity property was tested with several *ε* parameters and users’ anonymity was respected if (i) the geolocation data were aggregated by at least 5 by 5 for two distinct users and (ii) the designated perimeter was a circle of 5 km in diameter (Fig. 4) for urban zones. The circle perimeter would be automatically adjusted as needed by the algorithm to fit the first condition (aggregate at least 5 distinct data corresponding to at least 2 different users). If we reconsider the example of the “testers”, the algorithm could merge the data of another user to create a location zone big enough to merge the data of two distinct users. We used the same process with users having declared more than 30 VAS days or between 7 to 15 VAS days and showed that anonymization was found for all users. For users having declared only 1 day of VAS, it is mandatory to merge their geolocation data to at least one other user in order to de-identify their information. The algorithm could merge the one VAS day- user’s location with up to 5 users if they had all declared only 1 day of VAS. But even if the users declare only 1 day of VAS, the k-anonymity method allows the de-identification of the related results since all the results are aggregated to get a virtual position as the barycentre of the circle.

This method does not alter the initial quality of the VAS data but creates a location zone big enough to respect users’ privacy. When more users are identified in this area, the algorithm will be automatically adjusted to create a sharper location zone to fit the above condition.

Below is an example of k-anonymity applied to the users of Valenciennes (France). The circle is calculated to gather 5 data of at least 2 distinct users. This creates a circle of 2.5 km of radius that provides an artificial location at the centre of the circle for each data.

## Discussion

The present study in 5428 users from 22 countries showed that the precision of the geolocation data transferred by the smart phone is useful and reliable. The privacy of geolocation was evaluated by two methods, first on the five “testers” then on the 518 users declaring more than 30 days of VAS, and also in a sample of 234 users reporting 7 to 15 days of VAS. Special attention was also paid to users declaring VAS data only once. k-anonymity appeared to be relevant for data privacy of the *Allergy Diary*.

### Discussion of methods

The General Data Protection Regulation (GDPR) still recognizes quantification and gradation of anonymization methods.

For the *Allergy Diary* App, pseudonymizing cannot be considered as an anonymization technique because linking information data sets (such as pollen exposure) cannot guarantee that the initial sensitive data will not be recovered [23].

For random approaches (i) *Permutation* of data was not considered, as it would alter the quality of the database (DB); (ii) *Differential confidentiality* would imply the calculation of an aggregation estimator on sensitive data. We did not investigate this option since the DB could no longer be used to fit the MASK project objectives; (iii) *Noise addition* was tested. Using a zero value for the two last digits of the geolocation data, we were able to blur geolocation data in a 10 by 10 km area. Nevertheless, in our data set, some isolated users were still identifiable (Fig. 3).

Using k-anonymity, we tested several values of *ε* on our data set, and especially on the data collected for users registering 7 to 15 VAS days, these being our expected App user profiles. Users’ anonymization could always be obtained for a circle of 5 km in diameter. Interestingly, a 5 km circle would blur the localization data which is better than deleting the last two digits of the corresponding data in the noise addition approach (for example in Valenciennes as in Fig. 4). More generally, the algorithm can automatically adjust the radius of the circle when needed in order to fit the appropriate conditions (the k number of users and data).

We did not study any other generalization approaches. For instance, *l-diversity* [13] is an extension of the k-anonymity method but would imply the consideration of l distinct values, which is not possible in our data set. *t-proximity* [13] is even more stringent than the k-anonymity and l-diversity methods but we would need to know the general distribution of the sensitive data. This method would also imply the segregation of the data to obtain homogenous distribution classes. These data treatments would be too restrictive, and the overall DB quality would be affected.

The general strengths and weaknesses of the tools should be compared in terms of the three basic requirements proposed by WP29 [13] (Table 3).

**Table 3:** Strengths and weaknesses of anonymization tools (adapted from [13])

	Is singling out still a risk?	Is linkability still a risk?	Is inference still a risk?	Spoiling DB
Pseudonymisation	Yes	Yes	Yes	Yes
Noise addition	Yes	Yes	May not	May
k-anonymity(general)	No	May not	May not	No
k-anonymity on MASK-DB	No	No	No	No

k-anonymity applied to the MASK DB is sufficient to guarantee the users’ anonymity, not only on the current medical data set but also considering the integration of environmental data sets (e.g. pollen counts and pollution risks) yet to be gathered. No other DB containing personal data will be merged to our current data set in the future for the allergic rhinitis worldwide survey.

We therefore recommend the k-anonymization method (with our selected conditions/parameters) to anonymize this kind of geolocated medical data since this method does not interfere with the overall DB quality. This post treatment of sensitive data is an irreversible way of de-identifying the data collected through the App. The individualization of data is therefore respected, since even with k = 2, the probability of getting 5 days of identical VAS values is extremely low and, so far, has never been observed in our dataset. Considering the other data collected in our DB (such as the impact of allergic symptoms on daily activities), no correlation is possible with other data sets. Even if we integrate pollen counts and pollution risks, no personal data will be added to our database that could question the anonymization of our data set. Finally, interference (induction of sensitive information on any user) is impossible.

### Perspectives

Privacy of information is an increasing concern with the availability of large amounts of data from many individuals. In the *Allergy Diary* App, the mandatory data retrieved to use the app only include age, sex and country of living. This information is essential for adjusting the list of treatments available in the country of living. This is why the privacy concern has to focus only on geolocation data. In the future, we plan to apply our de-identification method, allowing us to merge our database with other sources of information that include precise geolocation data (for example: pollen and pollution exposition), while respecting users’ life privacy. These results are applicable to other DBs using geolocated data for any field of medicine.

The DB anonymization of “trajectories” (i.e. time and position information) will be considered as the number of users increases as well as the duration of the reporting. We will then consider anonymizing the data at the export phase (for analysis) with clusters of trajectories [34].

Guidelines are based on the assumption that patients regularly use their treatment and that recommendations are not tested with real-life data. Moreover, for many questions, recommendations are uncertain. Next-generation guidelines will need to use anonymized real-life data optimally retrieved using mobile technology to fill the current gaps. The results of this paper will then be used for guideline development.

## Conclusions

k-anonymity is an acceptable method for the anonymization of MASK data. It can also be used in other medical app-collected DBs in any fields of medicine. The remaining risk of identification is quite acceptable when considering the “reasonable means” [8, 31] used for re-identification with regards to the Recital 26 GDPR [11]. This k-anonymization method will be used for all data collected through the MASK project and this process will be written in the users’ legal document (“Terms of Use”/“Privacy Policy”). The post treatment of personal data is therefore considered to be compatible with the information given to the users when installing the App on their personal phone.

## Abbreviations

AR: Allergic rhinitis
ARIA: Allergic Rhinitis and its Impact on Asthma
DB: Data base
EU: European Union
GDPR: General data protection regulation
ICT: Information and communication technology
MACVIA: Contre les MAladies Chroniques pour un VIeillissement Actif et en bonne santé
MASK: MACVIA-ARIA Sentinel networK; Mobile Airways Sentinel networK
VAS: Visual analogue scale
